# Research on the Motion Control Strategy of a Lower-Limb Exoskeleton Rehabilitation Robot Using the Twin Delayed Deep Deterministic Policy Gradient Algorithm

**DOI:** 10.3390/s24186014

**Published:** 2024-09-17

**Authors:** Yifeng Guo, Min He, Xubin Tong, Min Zhang, Limin Huang

**Affiliations:** 1 School of Mechanical Engineering, Chengdu University, Chengdu 610106, China; guobujia2000@163.com (Y.G.);; 2 School of Artificial Intelligence, Hezhou University, Hezhou 542899, China

**Keywords:** lower-limb exoskeleton rehabilitation robot, motion trajectory planning and tracking, TD3 algorithm

## Abstract

The motion control system of a lower-limb exoskeleton rehabilitation robot (LLERR) is designed to assist patients in lower-limb rehabilitation exercises. This research designed a motion controller for an LLERR-based on the Twin Delayed Deep Deterministic policy gradient (TD3) algorithm to control the lower-limb exoskeleton for gait training in a staircase environment. Commencing with the establishment of a mathematical model of the LLERR, the dynamics during its movement are systematically described. The TD3 algorithm is employed to plan the motion trajectory of the LLERR’s right-foot sole, and the target motion curve of the hip (knee) joint is deduced inversely to ensure adherence to human physiological principles during motion execution. The control strategy of the TD3 algorithm ensures that the movement of each joint of the LLERR is consistent with the target motion trajectory. The experimental results indicate that the trajectory tracking errors of the hip (knee) joints are all within 5°, confirming that the LLERR successfully assists patient in completing lower-limb rehabilitation training in a staircase environment. The primary contribution of this study is to propose a non-linear control strategy tailored for the staircase environment, enabling the planning and control of the lower-limb joint motions facilitated by the LLERR.

## 1. Introduction

By the end of 2019, the global population of individuals aged 60 or above had reached 1 billion, with an estimated projection of 1.4 billion by 2030 [1]. The aging demographic has notably contributed to a rise in the number of stroke cases [2], with lower-limb motor dysfunction being a prevalent symptom among this group [3]. Additionally, lower-limb motor dysfunction ranks as the second most prevalent cause of mortality on a global scale [4], significantly affecting individual health [5]. Many stroke survivors need rehabilitation interventions to regain their mobility [6]. Nevertheless, the existing medical resources are insufficient [7]. The research and promotion of lower-limb exoskeleton rehabilitation robots (LLERRs) offer solutions to the disparity between supply and demand in rehabilitation medical resources, enhancing the quality of life for the elderly, and carrying substantial societal significance [8].

Lower-limb exoskeleton rehabilitation robotics is an interdisciplinary technology that integrates rehabilitation medicine, bionics, e-informatics, robotics, and mechanics to assist stroke patients in recovering their walking ability [9]. It aids in the restoration of lower-limb function and muscle strength, enhances balance and gait stability through tailored rehabilitation exercises [10,11]. Equipped with various sensors, the robot can continuously monitor the patient’s movement parameters in real time. This capability enables the evaluation of the patient’s progress and the delivery of individualized rehabilitation programs and guidance [12], thereby expediting the recovery process and enhancing its effectiveness [13].

Efforts have been undertaken by governmental bodies, businesses, scholars, and other stakeholders to work towards the objective of aiding a greater number of patients with lower-limb impairments in regaining motor functionality and advancing the utilization of lower-limb rehabilitation robotics.

Governments have been actively involved in promoting the research and development of rehabilitation robots since 2016. The Chinese government has implemented policies to encourage the advancement and utilization of rehabilitation robots [14]. Similarly, the Japanese government supports the development of assistive robots to enhance longevity and health by endorsing rehabilitation centers and fostering partnerships with medical research and development organizations [15]. The National Rehabilitation Center of Korea initiated the Translational Research Program for Rehabilitation Robotics. This program is designed to boost the growth of rehabilitation robotics and related industries by integrating technology-driven research and development with clinical applications [16].

Enterprises: Hocoma has introduced Andago, a mobile robot designed for lower-limb rehabilitation. This innovative device offers dynamic weight-bearing upright walking exercises along with features for fall prevention and real-time tracking [17]. Dai Ai Robotics has created AiWalker, a robot-assisted system for gait training that enhances the balance and walking capabilities of individuals recovering from strokes [18]. Shenzhen Maibu has developed BEAR-H1, a robotic system for lower-limb rehabilitation. Utilizing AI algorithms, it can anticipate the user’s movements and accommodate both active and passive training approaches. The robot assists patients in exercising their hip, knee, and ankle joints, facilitating comprehensive walking training [19].

Researchers: Glowinski, Sebastian et al. [20], in the field of inverse kinematics, proposed a redundant chain algorithm based on analytic equations. Chen Zhenlei et al. [21] introduced a control method for a lower-limb exoskeleton rehabilitation robot system that incorporated uncertain models. The approach utilized a deep Gaussian process for predicting motion intention and integrated a parameter for self-tuning the variable-conductance controller to enhance the system’s control strategy. Zhou Jie et al. [22] developed a trajectory deformation algorithm as a high-level trajectory planner to improve suppleness and motion smoothness in human–robot collaborative control. This algorithm planned the desired trajectory of a patient based on the interaction forces observed during physical human–robot interactions. Furthermore, Xu Jiajun et al. [23] presented a mirror therapy lower-limb rehabilitation robot designed for patients with a bias. They devised a motion generation scheme for the affected limb based on learning principles. The robot facilitated the active engagement of affected muscles by transferring movements from the healthy limb to the affected limb, encouraging imitation of the healthy limb’s movements.

According to the majority of scholarly investigations, advancements have been made in lower-limb rehabilitation robots; however, there are still deficiencies in specific areas. Current studies on environmental adaptability predominantly concentrate on level-ground terrains, whereas practical scenarios involve more intricate settings like stairs and inclines. Furthermore, although existing research considers the mechanical framework concerning patient dimensions, customized therapy requires further enhancement. In recent years, artificial intelligence technology has progressively been incorporated into the realm of robotics [24], equipping robots with enhanced cognitive, decision-making, and operational capabilities. This progression also presents greater potential for rehabilitation robots, which are expected to tackle current challenges and provide more accurate and individualized solutions for rehabilitation therapy.

Current scholarly investigations in the domain of artificial intelligence applied to lower-limb rehabilitation robots predominantly focus on two key areas: the utilization of machine learning technology for gait recognition and prediction in lower-limb movements, and the integration of reinforcement learning techniques to improve control strategies for lower-limb exoskeletons. Various innovative methodologies have been introduced by researchers for gait recognition and prediction. For instance, Li, Guoxin et al. [25] proposed a technique that employed Long Short-Term Memory networks to forecast the movement intentions of individuals by analyzing Surface Electromyography (sEMG) signals. This method enabled the activation of assistive devices to support rehabilitation patients during their training sessions. Furthermore, Lu, Yanzheng et al. [26] utilized stacked convolutional and Long Short-Term Memory networks to estimate joint angles (e.g., hip, knee, ankle) during activities such as walking, running, and stair climbing based on sEMG signals. Additionally, Zhang, Zaifang et al. [27] developed an integrated network model that combined a Sparse Autoencoder, a Bidirectional Long Short-Term Memory, and a Deep Neural Network to accurately identify different gait phases. Conversely, existing research also explores the integration of reinforcement learning into exoskeleton control to enhance the performance and adaptability of rehabilitation robots. For instance, Zhang, Qiang et al. [28] utilized a model-free reinforcement learning control framework to precisely adjust the standardized range of motion and gait patterns at the hip joint during walking, addressing the adaptability limitations associated with conventional methods. Additionally, Rose, Lowell et al. [29] proposed a new model free deep reinforcement learning method for learning gait patterns required for ground gait rehabilitation through exoskeletons. Furthermore, Li, Jingang et al. [30] addressed energy consumption challenges in lower-limb exoskeletons by implementing an energy management control strategy based on time-difference reinforcement learning. This strategy added a state-of-charge prediction model to effectively manage the energy consumption of the robots, thereby increasing their operational efficiency and sustainability in practical applications.

While AI algorithms have made significant progress in intent recognition and optimizing control algorithm parameters, there is a lack of research on the adaptability of rehabilitation robots in complex environments. This paper presents a motion controller for an LLERR using the TD3 algorithm to tackle motion control challenges in stair environments. The controller is designed to provide personalized rehabilitation treatment plans, improve the efficiency and safety of rehabilitation robots in real-world scenarios, and meet the diverse rehabilitation requirements of patients in different environments.

The main findings of this study include:

(1) The development of a trajectory planning controller for the LLERR, using the TD3 algorithm. This controller is responsible for generating the desired motion trajectory for the hip and knee joints of the LLERR.
(2) The development of a tracking controller for the LLERR using the TD3 algorithm. This controller aims to regulate the movement of the hip (knee) joint of the LLERR to follow a specified motion trajectory accurately, facilitating the execution of up-stairs movements.
(3) The study involves performing motion planning and tracking experiments using the LLERR to showcase the efficacy of the TD3 algorithm and the dependability of the LLERR control system, as supported by the experimental findings.

The subsequent sections of this paper are organized as follows: Section 2 elaborates on the mathematical model of LLERR. Section 3 outlines the controller’s design process and showcases its viability. Section 4 delineates the experimental procedures and outcomes, along with an extensive analysis of the results. Lastly, Section 5 presents the conclusion.

## 2. Mathematical Model

To investigate the motion control system of the LLERR, a lumped parameter mathematical model was developed in this study to characterize its dynamics. Before modeling, three assumptions were made to streamline the system description:

(1) The LLERR s’ exoskeleton was assumed to only move in the sagittal plane.
(2) The thigh (calf) was assumed to be concentrated at its center of point.
(3) We simplified the foot sole to a mass point, disregarding its specific shape.

### 2.1. Environmental Model

**Figure 1:**
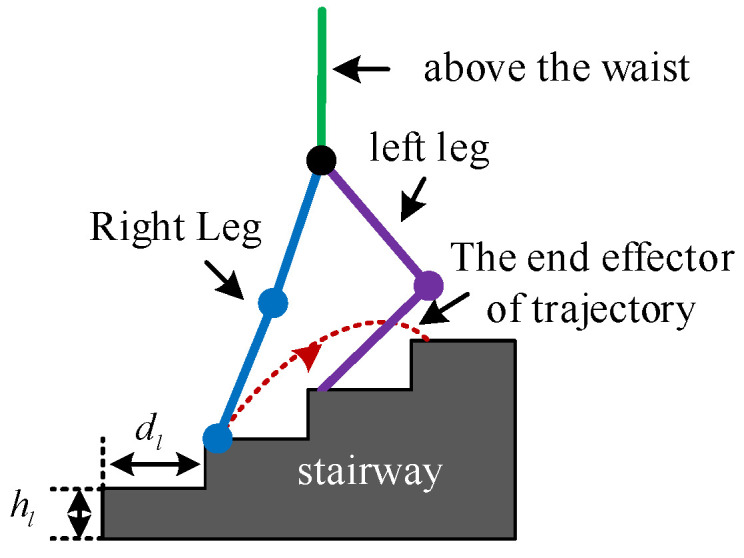
Operating environment of lower-limb exoskeleton rehabilitation robot.

Structured terrain is the most common type of terrain encountered in human daily life, comprising flat ground, stairs, and ramps. To ensure safe and smooth movement of an LLERR in these environments, it is crucial to plan a reasonable gait. This paper aimed to adapt the LLERR to the staircase environment by designing and planning the LLERR’s joint motion trajectories. Figure 1 shows the LLERR’s terrain environment, with a step height of $h_{l}$ and a depth of $d_{l}$.

### 2.2. Lower-Limb Exoskeleton Rehabilitation Robot Model

**Figure 2:**
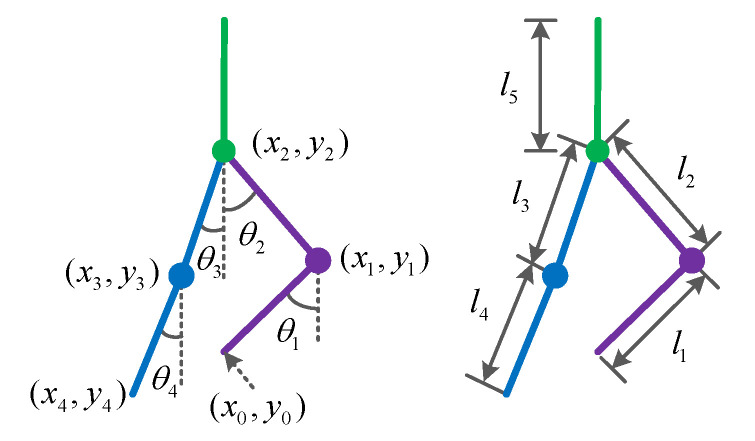
Lower-limb exoskeleton rehabilitation robot model.

Figure 2 shows the LLERR on a staircase, with the left leg on a higher step and the right leg on a lower step. In order to describe the robot posture, a five connecting rod model is used, and the angles and parameters are defined. The angle between the left shank and the vertical is $\theta_{1}$, the angle between the left thigh and the vertical is $\theta_{2}$, the angle between the right thigh and the vertical is $\theta_{3}$, and the angle between the right shank and the vertical is $\theta_{4}$. The angle is defined as 0 degrees when the thigh (shank) overlaps with the vertical. Clockwise rotation is considered positive, while counterclockwise rotation is considered negative. The left shank has a length of $l_{1}$ and a mass point of $m_{1}$. The left thigh has a length of $l_{2}$ and a mass point of $m_{2}$. The right thigh has a length of $l_{3}$ and a mass point of $m_{3}$. The right shank has a length of $l_{4}$ and a mass point of $m_{4}$. The upper torso has a length of $l_{5}$ and a mass point of $m_{5}$.

The dynamics equation of the LLERR is obtained from the Lagrange equation [31] as:

(1) $$F_{i}=\frac{d\left(\frac{\partial L}{\partial {\dot{\theta }}_{i}}\right)}{dt}-\frac{\partial L}{\partial \theta_{i}}\;\;i=1,2,3,4,$$

where F is the hip (knee) joint driving force; θ is the hip (knee) joint angle. $\dot{\theta }$ is the hip (knee) joint angular velocity; and L is the Lagrange function with the expression:

(2) $$L=E_{K}-E_{P},$$

where $E_{K}$ is the sum of the kinetic energy and $E_{P}$ is the sum of the potential energy of LLERR.

(3) $$E_{K}=\sum_{i=1}^{5}\left(\frac{1}{2}m_{i}({\dot{x}}_{i}^{2}+{\dot{y}}_{i}^{2})+\frac{1}{2}J_{i}{\dot{\theta }}_{i}^{2}\right),$$

(4) $$E_{p}=\sum_{i=1}^{5}m_{i}gy_{i},$$

where $\dot{x}$ is the velocity in the horizontal direction, $\dot{y}$ is the velocity in the vertical direction, J is the rotational inertia, and y is the vertical coordinate.

The coordinates of the left sole are $\left(x_{0},y_{0}\right)$, and the coordinates of the hip (knee) joint as well as the right sole are:

(5) $$\left\{\begin{array}{l} x_{i}=x_{i-1}-l_{i}\sin\theta_{i}\\ y_{i}=y_{i-1}+l_{i}\cos\theta_{i} \end{array}\right.,$$

Then, the Lagrange dynamical equation for LLERR is:

(6) $$F=D(\theta )\ddot{\theta }+H(\theta ,\dot{\theta })\dot{\theta }+G(\theta ),$$

where $\ddot{\theta }$ is the hip (knee) joint angular acceleration; D(θ) is the inertia matrix; $H(\theta ,\dot{\theta })$ is the centrifugal and kyphotic force matrix; G(θ) is the gravity matrix; and its expression is:

(7) $$F={\left[\begin{array}{cccc} F_{1} & F_{2} & F_{3} & F_{4} \end{array}\right]}^{T},$$

(8) $$\left\{\begin{array}{l} \theta ={\left[\begin{array}{cccc} \theta_{1} & \theta_{2} & \theta_{3} & \theta_{4} \end{array}\right]}^{T}\\ \dot{\theta }={\left[\begin{array}{cccc} {\dot{\theta }}_{1} & {\dot{\theta }}_{2} & {\dot{\theta }}_{3} & {\dot{\theta }}_{4} \end{array}\right]}^{T}\\ \ddot{\theta }={\left[\begin{array}{cccc} {\ddot{\theta }}_{1} & {\ddot{\theta }}_{2} & {\ddot{\theta }}_{3} & {\ddot{\theta }}_{4} \end{array}\right]}^{T} \end{array}\right.,$$

(9) $$D(\theta )=\left[\begin{array}{cccc} D_{11} & D_{12} & D_{13} & D_{14}\\ D_{21} & D_{22} & D_{23} & D_{24}\\ D_{31} & D_{32} & D_{33} & D_{34}\\ D_{41} & D_{42} & D_{43} & D_{44} \end{array}\right],$$

(9a) $$\left\{\begin{array}{l} D_{11}=\frac{1}{12}l_{1}^{2}m_{1}+\frac{1}{12}l_{2}^{2}m_{2}+\frac{1}{4}l_{1}^{2}m_{2}\cos^{2}\theta_{1}+l_{1}^{2}(m_{2}+m_{3}+m_{4}+m_{5})\sin^{2}\theta_{1}\\ D_{12}=\frac{1}{12}l_{2}^{2}m_{2}+l_{1}l_{2}(\frac{1}{2}m_{2}+m_{3}+m_{4}+m_{5})\sin\theta_{1}\sin\theta_{2}\\ D_{13}=-l_{1}l_{3}(\frac{1}{2}m_{3}+m_{4})\sin\theta_{1}\sin\theta_{3}\\ D_{14}=-\frac{1}{2}l_{1}l_{4}m_{4}\sin\theta_{1}\sin\theta_{4} \end{array}\;\;\right.,$$

(9b) $$\left\{\begin{array}{l} D_{21}=\frac{1}{12}l_{2}^{2}m_{2}+l_{1}l_{2}(\frac{1}{2}m_{2}+m_{3}+m_{4}+m_{5})\sin\theta_{1}\sin\theta_{2}\\ D_{22}=\frac{1}{12}l_{2}^{2}m_{2}+\frac{1}{12}l_{3}^{2}m_{3}+l_{2}^{2}(\frac{1}{4}m_{2}+m_{3}+m_{4}+m_{5})\sin^{2}\theta_{2}\\ D_{23}=\frac{1}{12}l_{3}^{2}m_{3}-l_{2}l_{3}(\frac{1}{2}m_{3}+m_{4})\sin\theta_{2}\sin\theta_{3}\\ D_{24}=-\frac{1}{2}l_{2}l_{4}m_{4}\sin\theta_{2}\sin\theta_{4} \end{array}\right.,$$

(9c) $$\left\{\begin{array}{l} D_{31}=-l_{1}l_{3}(\frac{1}{2}m_{3}+m_{4})\sin\theta_{1}\sin\theta_{3}\\ D_{32}=\frac{1}{12}l_{3}^{2}m_{2}-l_{2}l_{3}(\frac{1}{2}m_{3}+m_{4})\sin\theta_{2}\sin\theta_{3}\\ D_{33}=\frac{1}{3}l_{3}^{2}m_{3}+l_{3}^{2}m_{4}+\frac{1}{12}l_{4}^{2}m_{4}\\ D_{34}=-\frac{1}{12}l_{4}^{2}m_{4}+\frac{1}{2}l_{3}l_{4}m_{4}\cos(\theta_{3}-\theta_{4}) \end{array}\right.,$$

(9d) $$\left\{\begin{array}{l} D_{41}=-\frac{1}{2}l_{1}l_{4}m_{4}\sin\theta_{1}\sin\theta_{4}\\ D_{42}=-\frac{1}{2}l_{2}l_{4}m_{4}\sin\theta_{2}\sin\theta_{4}\\ D_{43}=-\frac{1}{12}l_{4}^{2}m_{4}-\frac{1}{2}l_{2}l_{3}m_{4}\cos(\theta_{3}-\theta_{4})\\ D_{44}=\frac{1}{3}l_{4}^{2}m_{4} \end{array}\right.,$$

(10) $$H(\theta ,\dot{\theta })=\left[\begin{array}{cccc} H_{11} & H_{12} & H_{13} & H_{14}\\ H_{21} & H_{22} & H_{23} & H_{24}\\ H_{31} & H_{32} & H_{33} & H_{34}\\ H_{41} & H_{42} & H_{43} & H_{44} \end{array}\right],$$

(10a) $$\left\{\begin{array}{l} H_{11}=l_{1}^{2}(\frac{3}{8}m_{2}+\frac{1}{2}m_{3}+\frac{1}{2}m_{4}+\frac{1}{2}m_{5})\sin2\theta_{1}\cdot {\dot{\theta }}_{1}\\ H_{12}=l_{1}l_{2}(\frac{1}{2}m_{2}+m_{3}+m_{4}+m_{5})\cos\theta_{2}\sin\theta_{1}\cdot {\dot{\theta }}_{2}\\ H_{13}=-l_{1}l_{3}(\frac{1}{2}m_{3}+m_{4})\cos\theta_{3}\sin\theta_{1}\cdot {\dot{\theta }}_{3}\\ H_{14}=-\frac{1}{2}l_{1}l_{4}m_{4}\cos\theta_{4}\sin\theta_{1}\cdot {\dot{\theta }}_{4} \end{array}\right.,$$

(10b) $$\left\{\begin{array}{l} H_{21}=l_{1}l_{2}(\frac{1}{2}m_{2}+m_{3}+m_{4}+m_{5})\cos\theta_{1}\sin\theta_{2}\cdot {\dot{\theta }}_{1}\\ H_{22}=l_{2}^{2}(\frac{1}{8}m_{2}+\frac{1}{2}m_{3}+\frac{1}{2}m_{4}+\frac{1}{2}m_{5})\sin2\theta_{2}\cdot {\dot{\theta }}_{2}\\ H_{23}=-l_{2}l_{3}(\frac{1}{2}m_{3}+m_{4})\cos\theta_{3}\sin\theta_{2}\cdot {\dot{\theta }}_{3}\\ H_{24}=-\frac{1}{2}l_{2}l_{4}m_{4}\cos\theta_{4}\sin\theta_{2}\cdot {\dot{\theta }}_{4} \end{array}\right.,$$

(10c) $$\left\{\begin{array}{l} H_{31}=l_{1}l_{3}(\frac{1}{2}m_{3}-m_{4})\cos\theta_{1}\sin\theta_{3}\cdot {\dot{\theta }}_{1}\\ H_{32}=l_{2}l_{3}(-\frac{1}{2}m_{3}-m_{4})\cos\theta_{2}\sin\theta_{3}\cdot {\dot{\theta }}_{2}\\ H_{33}=0\\ H_{34}=\frac{1}{2}l_{3}l_{4}m_{4}\sin(\theta_{3}-\theta_{4})\cdot {\dot{\theta }}_{4} \end{array}\right.,$$

(10d) $$\left\{\begin{array}{l} H_{41}=-\frac{1}{2}l_{1}l_{4}m_{4}\cos\theta_{1}\sin\theta_{4}\cdot {\dot{\theta }}_{1}\\ H_{42}=-\frac{1}{2}l_{2}l_{4}m_{4}\cos\theta_{2}\sin\theta_{4}\cdot {\dot{\theta }}_{2}\\ H_{43}=\frac{1}{2}l_{3}l_{4}m_{4}\sin(\theta_{4}-\theta_{3})\cdot {\dot{\theta }}_{3}\\ H_{44}=0 \end{array}\right.,$$

(11) $$G(\theta )=\left[\begin{array}{l} -gl_{1}(\frac{1}{2}m_{1}+m_{2}+m_{3}+m_{4}+m_{5})\sin\theta_{1}\\ -gl_{2}(\frac{1}{2}m_{2}+m_{3}+m_{4}+m_{5})\sin\theta_{2}\\ -gl_{3}(\frac{1}{2}m_{3}+m_{4})\sin\theta_{3}\\ \frac{1}{2}gl_{4}m_{4}\sin\theta_{4} \end{array}\right],$$

The LLERR system equation is:

(12) $$\left\{\begin{array}{l} \ddot{\theta }=-D^{-1}(\theta )H(\theta ,\dot{\theta })\dot{\theta }-D^{-1}(\theta )G(\theta )+D^{-1}(\theta )F\\ y=\theta \end{array}\right.,$$

## 3. Motion Trajectory Planning and Control for a Lower-Limb Exoskeleton Rehabilitation Robot

The LLERR for lower-limb exoskeleton exhibits alternating cyclic reciprocal movements of the left and right legs while climbing stairs. This paper focused on studying and analyzing a complete motion cycle. In a current cycle, the left sole remained stationary while the left leg moved from flexion to extension, and the right leg moved from a lower step to a higher step. Figure 1 above shows a schematic diagram of this process.

### 3.1. Motion Path Planning for the Right-Foot Sole

**Figure 3:**
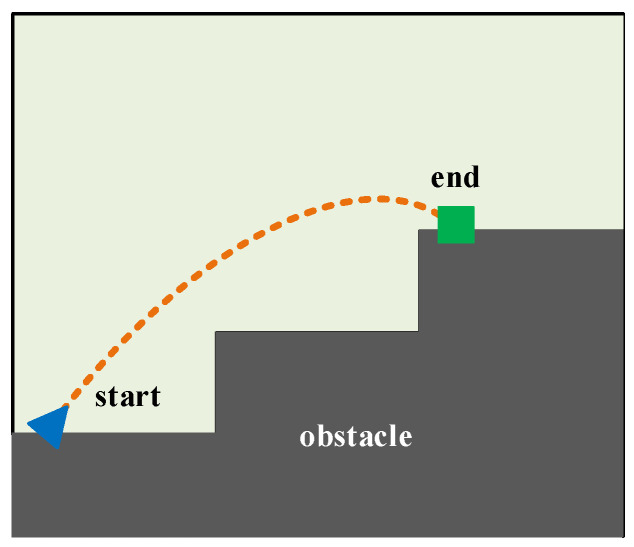
Gridding of the motion space of the mass point U.

The aim of this subsection was to plan a reasonable motion path for the mass point U located at the right-foot sole. Therefore, we focused solely on the right-foot sole of the LLERR and disregarded the other parts. To better analyze the motion path of the mass point U, we established a grid map, as shown in Figure 3. The map illustrates the path that the mass point U takes, departing from the start, avoiding obstacles, and reaching the end along a shorter and smoother path. To achieve this goal, we used the TD3 algorithm in this subsection.

**Figure 4:**
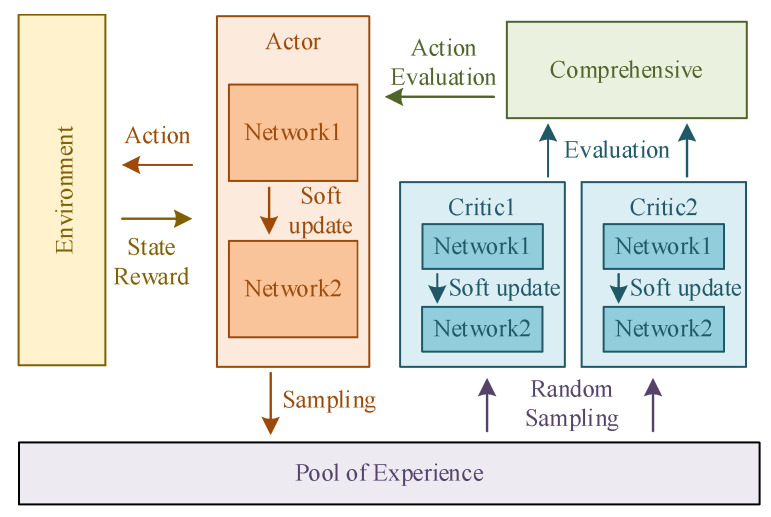
The motion path planning framework of mass point U.

Figure 4 shows the framework of the TD3 algorithm. Critic networks evaluate the state of the mass point U in the environment and determine the next action to be performed. Critic networks learn to improve the accuracy of their evaluations. Actor networks make decisions on the movement of the mass point and execute the strategy in the environment to generate data into the data pool. Actor networks also learn based on the evaluations of Critic networks.

Define the state of the mass point U at the moment t to be $S_{t}=(x_{t},y_{t})$, $x_{t}$ and $y_{t}$ are the coordinates of the mass point U at the moment t, and its spatial set is:

(13) $$S_{t}=\{(x_{t},y_{t})|0\leq x_{t}\leq x_{\max},0\leq y_{t}\leq y_{\max}\},$$

The action spatial set is:

(14) $$A(S_{t})=\{a_{t}|0{}^{\circ}\leq a_{t}<360{}^{\circ}\},$$

where $a_{t}$ is the direction chosen by the mass point U at moment *t*. The single-step reward $R_{t}$ represents the reward obtained by the mass point U for performing the action $a_{t}$ in the current state $S_{t}$:

(15) $$R_{t}(S_{t},a_{t})=\{r_{1},r_{2},r_{3}\},$$

where $r_{1}$ denotes the reward value for the next step to reach the end; $r_{2}$ is the reward value when the next step is a boundary or an obstacle; and $r_{3}$ is the reward value when the next step is not the end, an obstacle or a boundary.

The accumulated reward *Rn* represents the sum of the rewards earned by the mass point U for all the steps from start to end.

(16) $$Rn=\sum_{t=0}^{n}\gamma^{t}R(S_{t},a_{t})\;\left(0<\gamma <1\right),$$

where γ is the discount factor used to constrain the mass point U to take the long route.

To find a path that maximizes the accumulated reward over multiple learning sessions, the TD3 algorithm is used.

(17) $$\max{\left\{Rn_{i}\right\}}_{i=1}^{n},$$

**Figure 5:**
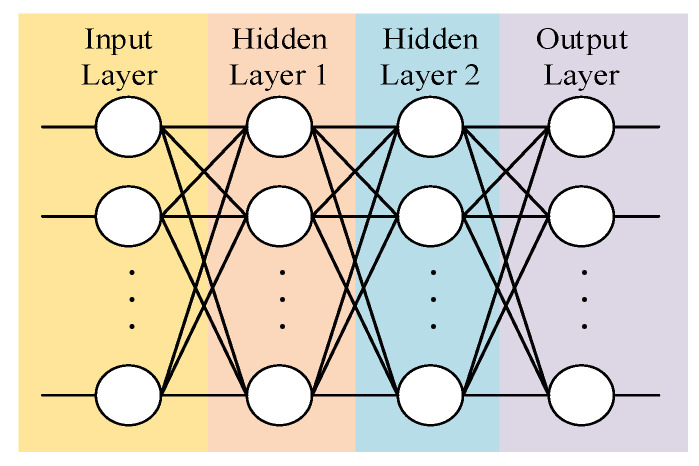
Critic (Actor) neural network topology.

Defining a Critic network whose network topology is shown in Figure 5, the input is the state $S_{t}$ and action $a_{t}$ of the mass point U. The hidden layers 1 and 2 take 128 neurons, respectively, and the activation function adopts the Relu function. The output is the value evaluated on the input data.

Defining an Actor network whose network topology is shown in Figure 5, the input is the state $S_{t}$ of the mass point U. The hidden layers 1 and 2 take 128 neurons, respectively, and the activation function adopts the Relu function. The output is the next actor.

Define the data pool for storing the planning trajectories of the mass point U. Define the synthesis module for synthesizing the Critic1 and Critic2 ratings to obtain the final action rating.

Critic network learning process: first, sample $S_{t}$, $R_{t}$, $S_{t+1}$ and calculate the TD target:

(18) $$y_{t}=R_{t}+\gamma v(S_{t+1},w),$$

where $v(S_{t+1},w)$ is the Critic network’s evaluation value for state $S_{t+1}$, and w is the Critic network parameter.

Then, the TD error $\delta_{t}$ is calculated as:

(19) $$\begin{array}{cc} \delta_{t} & =v(S_{t},w)-y_{t}\\ & =v(S_{t},w)-[R_{t}+\gamma v(S_{t+1},w)] \end{array},$$

Finally, the Critic network is trained using a gradient descent method:

(20) $$w=w-\alpha \delta_{t}\frac{\partial v(S_{t},w)}{\partial w},$$

where *α* is the learning rate.

The Actor network is trained using a gradient ascent method:

(21) $$\lambda =\lambda -\beta \delta_{t}\frac{\partial \ln\pi (a_{t}|S_{t},\lambda )}{\partial \lambda },$$

where *λ* is the Actor network parameters, $\pi (a_{t}|S_{t},\lambda )$ is the decision made by the Actor network, and *β* is the learning rate.

To improve the astringency of Critic and Actor networks, during the network learning process, the dual network delay soft update is used.

The pseudocode for planning the path of the mass point U is as follows Algorithm 1:
**Algorithm 1 Planning the path of the point U**1$\mathrm{Initialize}\;\mathrm{start}\;S_{0}$$,\;\mathrm{and}\;\mathrm{end}\;S_{e}$2$\mathrm{Initialize}\;\mathrm{Critic}\;\mathrm{network}’s\;v(w_{1})$$,\;v(w_{2})$, and Actor network’s π(λ)$,\;\mathrm{with}\;\mathrm{random}\;\mathrm{parameters}\;w_{1}$$,\;{w^{\prime }}_{1}$$,\;w_{2}$$,\;{w^{\prime }}_{2}$λ3$\mathrm{Initialize}\;\mathrm{target}\;\mathrm{network}’s\;v({w^{\prime }}_{1})$$,\;v({w^{\prime }}_{2})$$,\;\pi (\lambda^{\prime })$$,\;\mathrm{with}\;{w^{\prime }}_{1}\;=\;w_{1}$$,\;{w^{\prime }}_{2}\;=\;w_{2}$$,\;\lambda^{\prime }\;=\;\lambda$4$\mathrm{Initialize}\;\mathrm{sample}\;\mathrm{buffer}\;B_{u}$5**for** *k =* 1 **to** *K* **do**6  Actor network’s π(λ)
$\;\mathrm{makes}\;\mathrm{decisions},\;\mathrm{plans}\;a\;\mathrm{batch}\;\mathrm{of}\;\mathrm{paths}\;\mathrm{to}\;\mathrm{the}\;\mathrm{end}\;\mathrm{point},\;\mathrm{and}\;\mathrm{updates}\;\mathrm{the}\;\mathrm{buffer}\;B_{u}$
7  $\mathrm{Sample}\;N\;\mathrm{paths}\;\mathrm{from}\;\mathrm{buffer}\;B_{u}$
$\;\mathrm{and}\;\mathrm{obtain}\;S_{t}$
$,\;R_{t}$
$,\;S_{t+1}$
8  $\mathrm{The}\;\mathrm{TD}\;\mathrm{target}\;\mathrm{and}\;\mathrm{TD}\;\mathrm{error}\;\mathrm{are}\;\mathrm{calculated}\;\mathrm{through}\;\mathrm{the}\;\mathrm{target}\;\mathrm{network}’s\;v({w^{\prime }}_{1})$$\;\mathrm{and}\;v({w^{\prime }}_{2})$ by Equations (18) and (19)9  $\mathrm{Critic}\;\mathrm{network}’s\;v(w_{1})$$\;\mathrm{and}\;v(w_{2})$ are learned through Equation (20)10  Actor network’s π(λ) is learned through Equation (21)11  **if** *k* mod *d*, **then**12    $\mathrm{Update}\;\mathrm{target}\;\mathrm{network}’s\;v({w^{\prime }}_{1})$
$,\;v({w^{\prime }}_{2})$
$,\;\pi (\lambda^{\prime })$
13    $\lambda^{\prime }=\tau_{1}\lambda +(1-\tau_{1})\lambda^{\prime }$
14    ${w^{\prime }}_{1}=\tau_{2}w_{1}+(1-\tau_{2}){w^{\prime }}_{1}$
15    ${w^{\prime }}_{2}=\tau_{3}w_{2}+(1-\tau_{3}){w^{\prime }}_{2}$
16  **end if**
17**end for**18$\mathrm{Execute}\;\mathrm{the}\;\mathrm{Actor}\;\mathrm{network}\;\mathrm{decision}\;\mathrm{to}\;\mathrm{obtain}\;\mathrm{the}\;\mathrm{path}\;\mathrm{from}\;\mathrm{the}\;\mathrm{start}\;S_{0}$$\;\mathrm{to}\;\mathrm{the}\;\mathrm{end}\;S_{e}$

**Figure 6:**
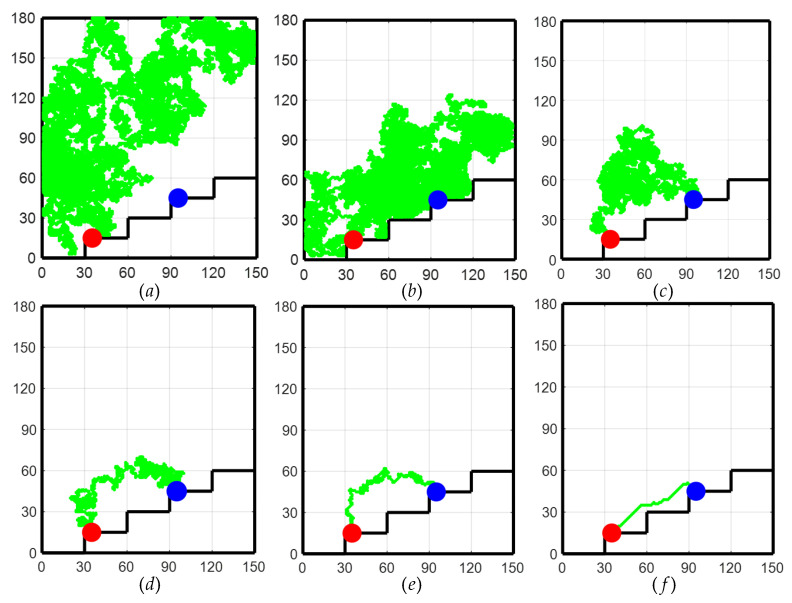
Path planning process for the TD3 algorithm.

As shown in Figure 6 for the path planning process of the TD3 algorithm, initially, due to the lack of a priori knowledge, only the global exploration strategy could be used. This resulted in the LLERR struggling to find the end point, as shown in Figure 6a. However, with the passage of time and the accumulation of experience from a series of failures, the system gradually learned an effective path planning method and eventually succeeded in finding the end point, as shown in Figure 6b. After accumulating successful experiences, the system gradually optimized the planning of the movement path, resulting in shorter and more reasonable paths as shown in Figure 6c–e. After 2000 iterations of learning, the algorithm successfully planned a shorter, smoother, and more reasonable motion path. This path could initially be considered a target curve for subsequent LLERR control. It provided feasibility and guidance for the system to exhibit superior motion planning in applications. The result is shown in Figure 6f.

**Remark 1.**  *In Figure 6, the red dots represent the starting point, the blue dots represent the target point, and the green dots represent the trajectory planned by the algorithm.*

**Figure 7:**
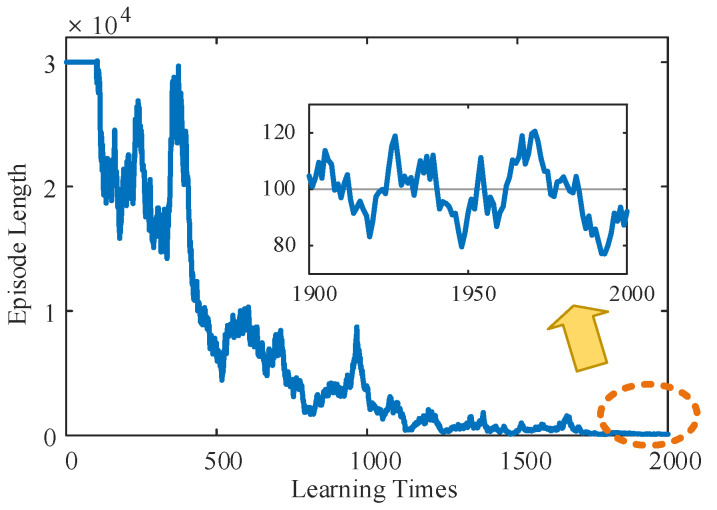
The relationship between the planning path length and learning times of mass point U.

According to the learning stage of the TD3 algorithm shown in Figure 7, the relationship between the planning path length and learning times of mass point U, in the initial stage, due to the lack of experience, was not able to find the end point in 30,000 runs, which meant that the system was in the blind exploration stage and needed to carry out a wide exploration of the whole map. However, once the end point was successfully located, the LLERR began to accumulate experience in reaching it and entered the stage of searching for the optimal path. As successes accumulated, the length of planned paths tended to decrease. After approximately 2000 learnings, the length of the planned path stabilized at around 100 processes. This shows that the accumulation of experience and the optimization of the system make the search for the optimal path more efficient and stable.

**Remark 2.**  *In the previous paragraph, the TD3 algorithm reached a steady state after the 100th iteration and successfully planned the target trajectory of the right foot. Subsequently, the LLERR system followed this target trajectory and moved from the start point to the end point, thus assisting the patient to complete a full stride.*

**Figure 8:**
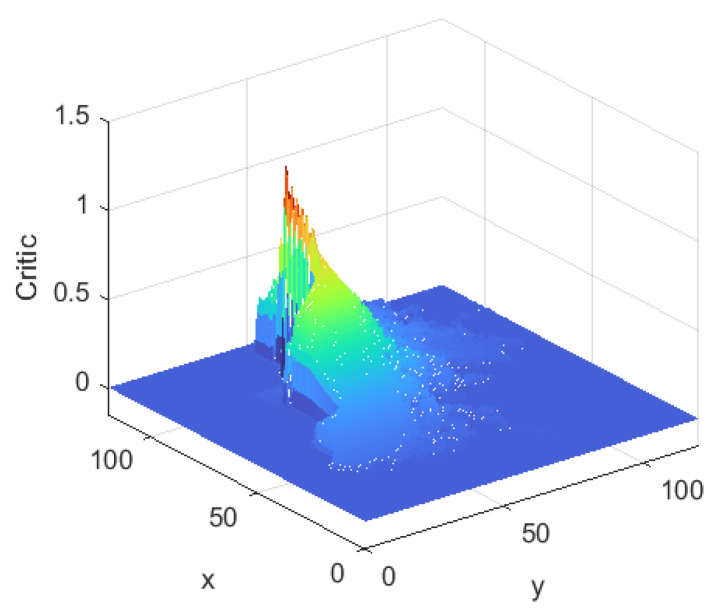
Critic of TD3 algorithm.

Figure 8 shows the evaluation system of the Critic after learning. It is red near the end point, with an evaluation value close to 1. In the color order of red, orange, yellow, green, green and blue, the evaluation value gradually decreases and eventually approaches 0. The rule that the farther away from the end point, the lower the evaluation value, clearly reflects the design principle of the evaluation system. The left side of the end point represents the inside of the staircase and therefore has a lower evaluated value in this area. Overall, the color distribution of the evaluation system was consistent with the characteristics of the environment and accurately reflected the evaluation values of different locations.

**Figure 9:**
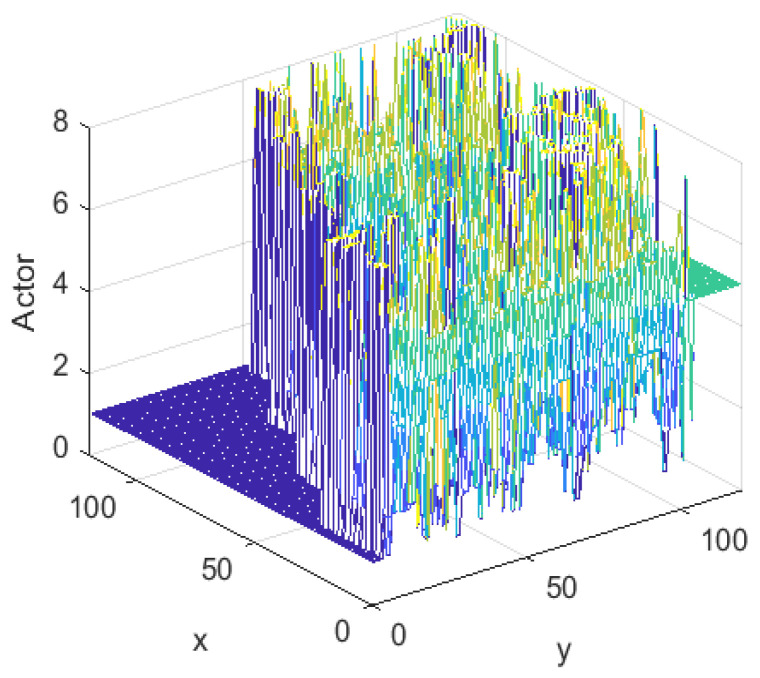
Actor of TD3 algorithm.

Figure 9 illustrates the Actor decision system that was learned. The left side of the figure represents the staircase region, which has a decision value of 90°. The colors, in order of purple, blue, cyan, green, yellow and orange correspond to different degrees of decision values between 90° and −270°, respectively. This decision system presented a reasonable distribution that matched the characteristics of the environment.

**Figure 10:**
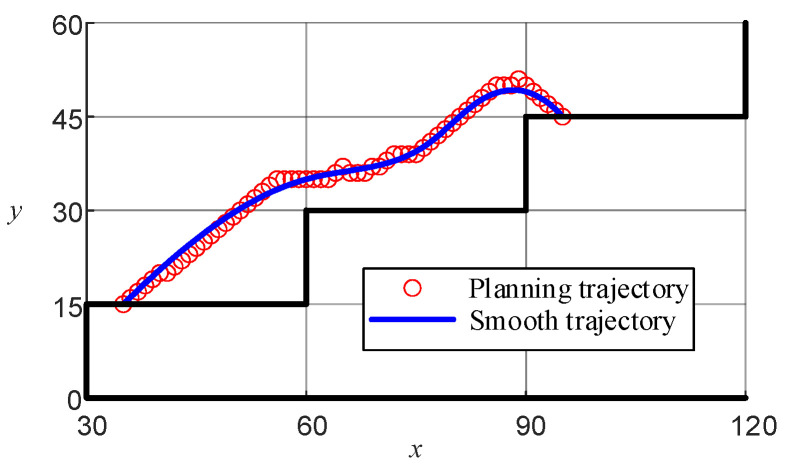
Lower-limb exoskeleton right-sole target path.

Figure 6f demonstrates that while the planning path could successfully navigate from the start to the end points, it lacked smoothness, which may negatively affect the motion control of the LLERR. To improve this, the cubic spline interpolation method was used to achieve a smoother planning path. The functional expression is shown in Equation (22), and the function plot is shown in Figure 10.

(22) $$y=\left\{\begin{array}{ll} -0.000195{\left(x-35\right)}^{3}-0.0094{\left(x-35\right)}^{2}+1.16(x-35)+15 & \left(35\leq x<52\right)\\ -0.000195{\left(x-52\right)}^{3}-0.0194{\left(x-52\right)}^{2}+0.67(x-52)+31 & \left(52\leq x<60\right)\\ 0.0015{\left(x-60\right)}^{3}-0.024{\left(x-60\right)}^{2}+0.32(x-60)+35 & \left(60\leq x<69\right)\\ 0.002{\left(x-69\right)}^{3}+0.0157{\left(x-69\right)}^{2}+0.2441(x-69)+37 & \left(69\leq x<77\right)\\ -0.0076{\left(x-77\right)}^{3}+0.0646{\left(x-77\right)}^{2}+0.8863(x-77)+41 & \left(77\leq x<83\right)\\ -0.000967{\left(x-83\right)}^{3}-0.0723{\left(x-83\right)}^{2}+0.84(x-83)+47 & \left(83\leq x<92\right)\\ -0.000967{\left(x-92\right)}^{3}-0.0984{\left(x-92\right)}^{2}-0.696(x-92)+48 & \left(92\leq x<95\right) \end{array}\right.,$$

### 3.2. Calculation of Angular Curves of Lower-Limb Motion Joints

The trajectories of the four joints of the left hip, left knee, right hip, and right knee can be deduced from the path of the right sole and the inverse kinematic equations of the LLERR. The expression is:

(23) $$\theta_{i}=\arctan\frac{x_{i}-x_{i-1}}{y_{i}-y_{i-1}}\;\;(i=1,2,3,4),$$

**Figure 11:**
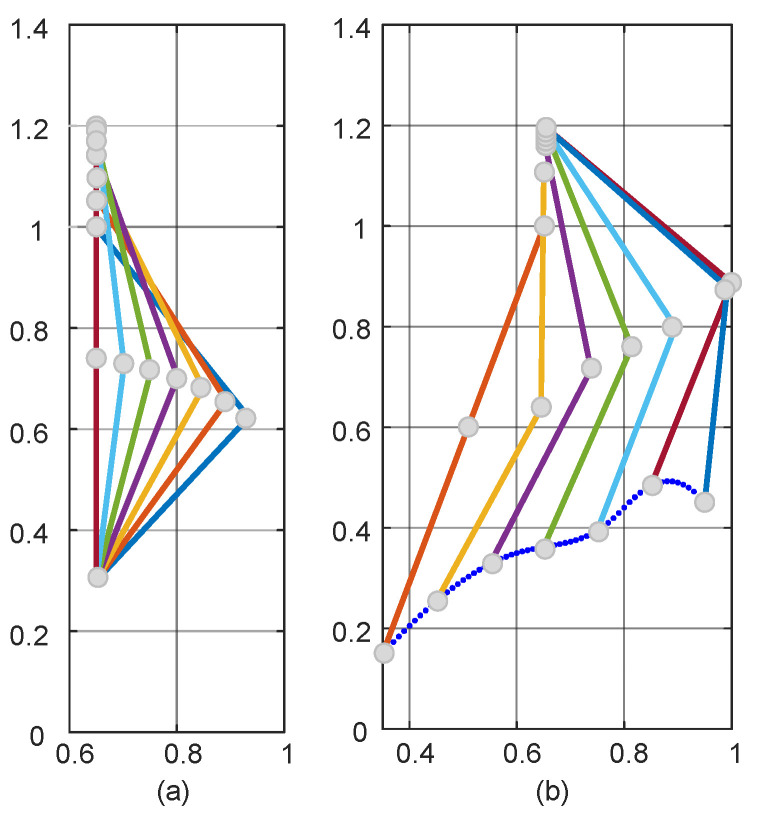
Schematic diagram of lower-limb exoskeleton’s single-leg movement.

Figure 11 shows the schematic diagram of LLERR completing a stride. One cycle was 2 s. Figure 11a shows the movement process of the left leg, where the left sole’s position remains constant, and the knee contracts backward while the hip extends upward. Figure 11b shows the movement process of the right leg, where the sole is on the ground, and the leg is straight. Then, the sole gradually leaves the ground, and the knee starts to bend forward while the hip extends upward. The right and left legs move together to complete a single stride cycle.

**Figure 12:**
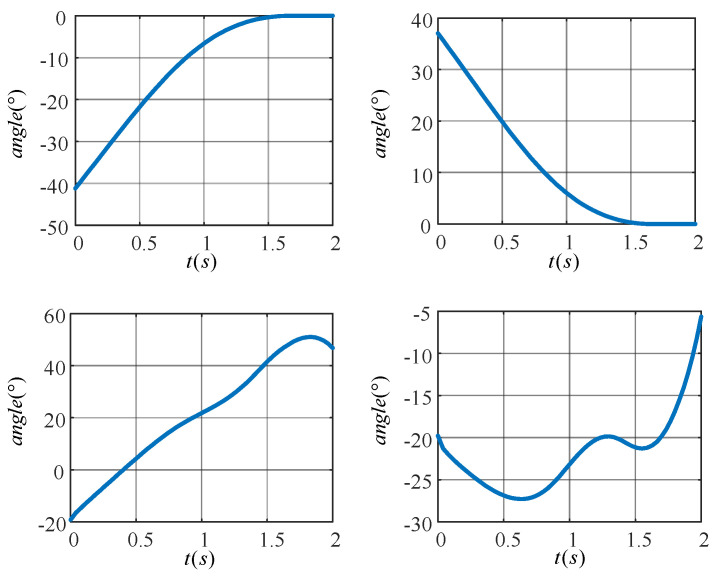
Target motion trajectories of the joints of a lower extremity exoskeleton rehabilitation robot.

In Figure 12a,b, the target motion trajectories of the knee (hip) joint of the left leg are shown. The angle of the knee joint gradually increases to zero degrees while the angle of the hip joint gradually decreases to zero degrees as the left leg is shifted from a flexed state to an upright state. Figure 12c,d show the target motion trajectories of the knee (hip) joint of the right leg, which completes the movement up the stairs. The angle of the hip joint tends to increase, while the angle of the knee joint increases in a spiral fashion.

Compared with Habib Mohamad et al.’s paper [32], the lower-limb target motion trajectory planned by the method in this paper is smoother. This has a positive impact on the subsequent joint motor control process. At the same time, the smoother motion trajectory design is more in line with the law of natural human movement, which effectively reduces the patient’s discomfort and potential risk of injury during the rehabilitation training process.

### 3.3. Motion Trajectory Tracking Control of a Lower-Limb Exoskeleton Rehabilitation Robot

Motion control of the LLERR is a continuous output problem, so in this subsection, we adopted the use of the continuous output TD3 algorithm. The algorithm’s framework aligns with the depiction in Figure 4 as previously discussed.

Define the hip (knee) joint angle of the LLERR to be S(t) at moment *t*. The state space is continuous, and the space set is:

(24) $$S(t)=\{(\theta_{1}(t),\theta_{2}(t),\theta_{3}(t),\theta_{4}(t)|\theta_{\min}\leq \theta_{i}(t)\leq \theta_{\max},\;\;\;i=1,2,3,4\},$$

Define the hip (knee) joint drive of the LLERR as action A(t) with a continuous action space, and space set A(t) is:

(25) $$A(t)=\{(F_{1}(t),F_{2}(t),F_{3}(t),F_{4}(t)|F_{\min}\leq F_{i}(t)\leq F_{\max},\;\;i=1,2,3,4\},$$

Define the single-step reward function as:

(26) $$R(t)=\sum_{i=1}^{4}\frac{1}{\left|\theta_{aim}-\theta_{i}\right|},$$

Define the accumulated reward, the sum of all rewards earned by the LLERR after performing action A(t) as

(27) $$Rn=\int_{0}^{2}R(t)dt,$$

The goal of using the TD3 algorithm for LLERR motion control is to find the appropriate F(t) over multiple learning sessions that allows the LLERR to track the planned trajectory, thus obtaining the maximum accumulated reward

(28) $$\max{\left\{Rn_{i}\right\}}_{i=1}^{n},$$

Define a Critic network; its network topology is shown in Figure 5. The input is the joints’ angle θ and joints’ drive F(t) of the LLERR. The hidden layers 1 and 2 take 128 neurons, respectively, and the activation function adopts the Relu function. The output is the value of evaluating the input data.

Define an Actor network, whose network topology is shown in Figure 5 above; the input is the joints’ angle $S_{t}$ of the LLERR. The hidden layers 1 and 2 take 128 neurons, respectively, and the activation function adopts the Relu function. The output is the joints drive A(t).

Define the data pool for storing the joints’ angle motion trajectory of the LLERR. Define the synthesis module for synthesizing the Critic1 and Critic2 ratings to obtain the final action rating.

The pseudocode for the TD3 algorithm to control the motion of the LLERR is as follows Algorithm 2:
**Algorithm 2 Motion control of a lower-limb exoskeleton rehabilitation robot**1$\mathrm{Initialize}\;\mathrm{left}\;\mathrm{knee}\;\theta_{1}$$,\;\mathrm{left}\;\mathrm{hip}\;\theta_{2}$$,\;\mathrm{right}\;\mathrm{hip}\;\theta_{3}$$,\;\mathrm{right}\;\mathrm{knee}\;\theta_{4}$ initial and target angles.2$\mathrm{Initialize}\;\mathrm{Critic}\;\mathrm{network}’s\;v(w_{1})$$\;\mathrm{and}\;v(w_{2})$, and Actor network’s π(λ)$,\;\mathrm{with}\;\mathrm{random}\;\mathrm{parameters}\;w_{1}$$,\;{w^{\prime }}_{1}$$,\;w_{2}$$,\;{w^{\prime }}_{2}$, λ3$\mathrm{Initialize}\;\mathrm{target}\;\mathrm{network}’s\;v({w^{\prime }}_{1})$$,\;v({w^{\prime }}_{2})$$,\;\pi (\lambda^{\prime })$$,\;\mathrm{with}\;{w^{\prime }}_{1}\;=\;w_{1}$$,\;{w^{\prime }}_{2}\;=\;w_{2}$$,\;\lambda^{\prime }\;=\;\lambda$4$\mathrm{Initialize}\;\mathrm{sample}\;\mathrm{buffer}\;B_{u}$5**for** *k =* 1 **to** *K* **do**6  The decisions F(t)
 produced by the Actor network’s π(λ)
$\;\mathrm{are}\;\mathrm{utilized}\;\mathrm{to}\;\mathrm{control}\;\mathrm{the}\;\mathrm{lower}\text{-}\mathrm{limb}\;\mathrm{exoskeleton}\;\mathrm{rehabilitation}\;\mathrm{robot}\;\mathrm{and}\;\mathrm{achieve}\;\mathrm{the}\;\mathrm{desired}\;\mathrm{joint}\;\mathrm{angles},\;\mathrm{as}\;\mathrm{well}\;\mathrm{as}\;\mathrm{update}\;\mathrm{the}\;\mathrm{buffer}\;B_{u}$
7  $\mathrm{Sample}\;N\;\mathrm{paths}\;\mathrm{from}\;\mathrm{buffer}\;B_{u}$ and obtain 0$S_{t}$$,\;R_{t}$$,\;S_{t+1}$
8  $\mathrm{The}\;\mathrm{TD}\;\mathrm{target}\;\mathrm{and}\;\mathrm{TD}\;\mathrm{error}\;\mathrm{are}\;\mathrm{calculated}\;\mathrm{through}\;\mathrm{the}\;\mathrm{target}\;\mathrm{network}’s\;v({w^{\prime }}_{1})$$\;\mathrm{and}\;v({w^{\prime }}_{2})$ by Equations (18) and (19)9  $\mathrm{Critic}\;\mathrm{network}’s\;v(w_{1})$$\;\mathrm{and}\;v(w_{2})$ are learned through Equation (20)10  Actor network’s π(λ) is learned through Equation (21)11  **if** *k* mod *d*, **then**12    $\mathrm{Update}\;\mathrm{target}\;\mathrm{network}’s\;v({w^{\prime }}_{1})$
$,\;v({w^{\prime }}_{2})$
$,\;\mathrm{and}\;\pi (\lambda^{\prime })$
13    $\lambda^{\prime }=\tau_{1}\lambda +(1-\tau_{1})\lambda^{\prime }$
14    ${w^{\prime }}_{1}=\tau_{2}w_{1}+(1-\tau_{2}){w^{\prime }}_{1}$
15    ${w^{\prime }}_{2}=\tau_{3}w_{2}+(1-\tau_{3}){w^{\prime }}_{2}$
16  **end if**
17**end for**18Decisions were generated by the Actor network’s π(λ) based on the starting joint angle. The decisions F(t) were inputted into the lower-limb exoskeleton rehabilitation robot to enable it to accurately track the target motion trajectory shown in Figure 12. The patient’s lower limb was successfully trained to climb the stairs in a single cycle.

**Figure 13:**
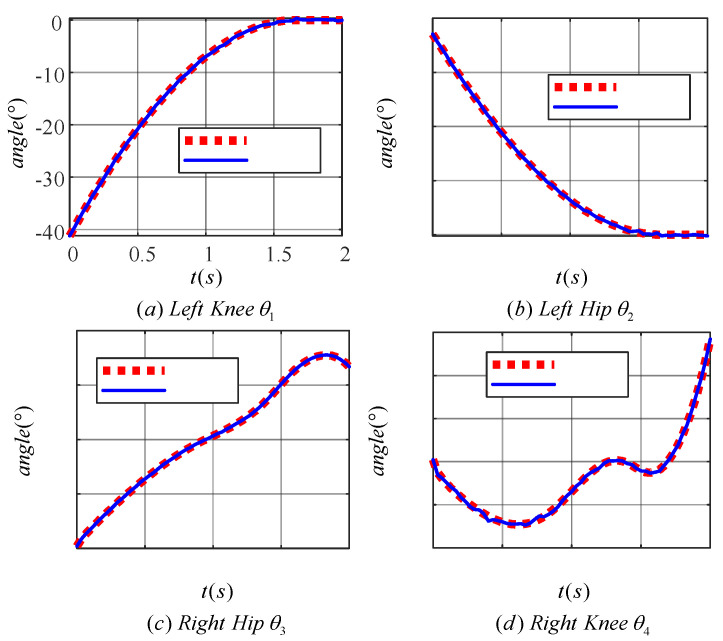
Joint angle tracking curves of a lower-limb exoskeleton rehabilitation robot.

Figure 13 demonstrates the effect of the learned TD3 algorithm on the control of LLERR. Subplots (a) and (b) present the angle tracking curves of the left leg hip and knee joints, while (c) and (d) show the angle tracking curves of the right leg’s hip and knee joints. In these curves, the red dotted line indicates the target joint angle, while the blue solid line indicates the output curve of the actual joint angle of the LLERR under the action of the TD3 algorithm. By observing these plots, it was found that the red dotted line and the blue solid line trended in basically the same way. This indicated that the actual output angle of the LLERR under the action of the TD3 algorithm could effectively track the target trajectory, which made the LLERR meet the needs of patients’ lower-limb rehabilitation in the staircase environment.

**Figure 14:**
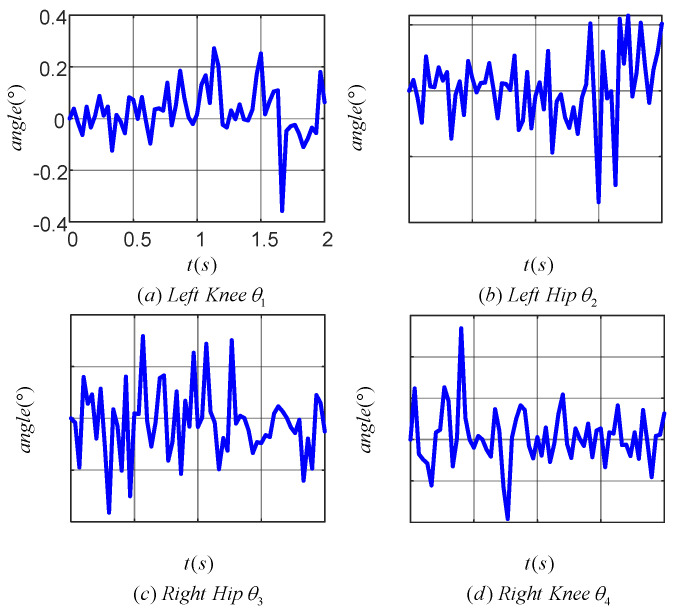
Joint angle tracking error curves of a lower-limb exoskeleton rehabilitation robot.

Figure 14 illustrates the tracking error of the TD3 algorithm for controlling the angle of the lower limb joints. Plots (a), (b), (c), and (d) correspond to the tracking errors of the left knee, left hip, right hip and right knee joints, respectively. The maximum tracking error was within 0.4° for the left knee, 0.4° for the left hip, 0.2° for the right hip, and 0.6° for the right knee. The results indicated that the TD3 algorithm was effective in reducing the angular tracking error of each joint of the LLERR. This demonstrated the TD3 algorithm’s efficacy from an error analysis perspective.

**Figure 15:**
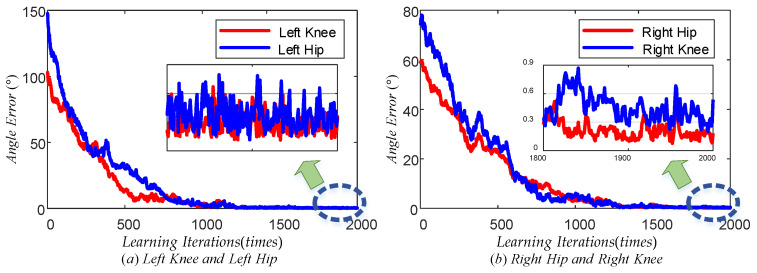
Maximum joint angle error vs. learning iterations.

Figure 15 illustrates the maximum tracking error trend of the TD3 algorithm for the left knee, left hip, right hip, and right knee during the learning phase. The red line represents the maximum tracking error of the left knee, while the blue line represents the maximum tracking error of the left hip in plot (a). Similarly, the red line represents the maximum tracking error of the right hip, while the blue line represents the maximum tracking error of the right knee in plot (b). Observing these plots, it can be concluded that all four curves exhibited a decreasing trend from a global perspective. This indicated that the maximum tracking error of each joint angle gradually decreased as the number of learning times increased. From a local perspective, the trend of decreasing the maximum tracking error for each joint became slow once the number of learning times reached a specific threshold. Specifically, following 1800 learning sessions, the maximum error was stabilized at approximately 0.4° for the left knee, 0.5° for the left hip, 0.2° for the right hip, and 0.6° for the right knee. When the number of learning iterations was less, the blue line in both (a) and (b) plots showed an increasing trend, which indicated that the learning direction of the left hip joint and the right knee joint was not accurate enough at the beginning of learning due to the lack of experience, but with the accumulation of experience, the learning direction was gradually adjusted to the correct direction. The above results demonstrated that the TD3 algorithm could effectively reduce the error of the LLERR after multiple learning iterations. This proved the effectiveness of the TD3 algorithm from the learning process.

## 4. Prototype Experiment of Lower-Limb Exoskeleton Rehabilitation Robot

**Figure 16:**
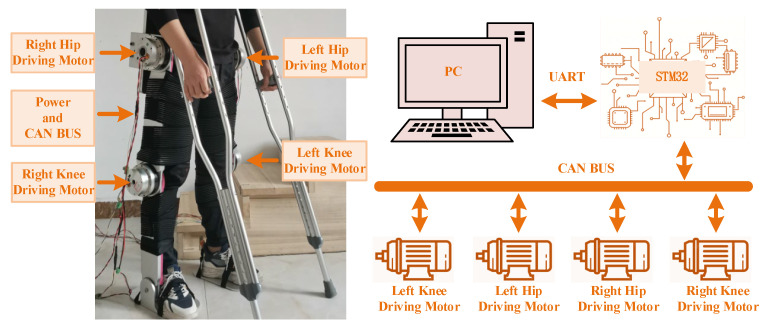
Experimental prototype testing platform for lower-limb exoskeleton rehabilitation robot.

Figure 16 shows the experimental prototype test platform of the LLERR. We invited a volunteer with a height of 165 cm, weight of 65 kg, shank length of 43 cm, and thigh length of 47 cm to climb a staircase with a height of 15 cm per step in the experimental environment. The parameters of each drive motor of the LLERR were as follows: the hip joint drive motor had a rated speed of 27 PRM, a rated torque of 133 N∙M, and a peak torque of 194 N∙M; the knee joint drive motor had a rated speed of 35 PRM, a rated torque of 107 N∙M, and a peak torque of 169 N∙M. The encoder resolution was 17 bits. The entire prototype platform was powered by a 48 V supply voltage, and the CAN bus was used for communication. The bottom motor drive control core was an STM32F407, and the upper TD3 algorithm running platform was a PC (Intel i7).

The trained model from the previous section was applied to the LLERR prototype testbed. It was used to plan the trajectory of the right sole and determine the motion curve of the target hip (knee) joint angle through backpropagation. Subsequently, the TD3 algorithm was used to control the joints of the LLERR to track the target curve. Finally, we observed the exercise effect of the LLERR and evaluated its performance and effectiveness in rehabilitation tasks.

**Figure 17:**
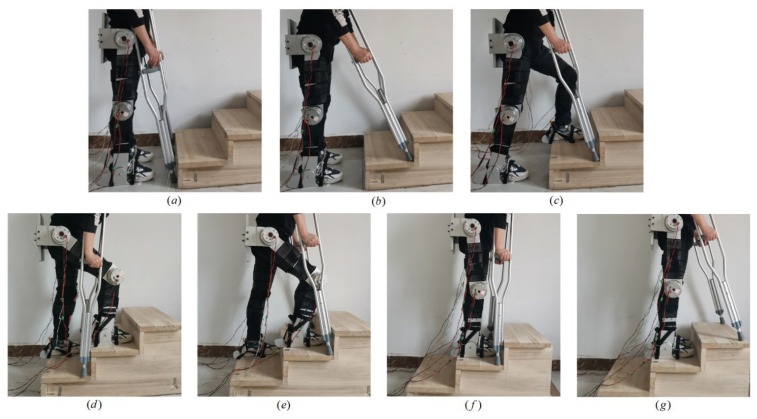
Prototype control system testing process of lower-limb rehabilitation robot.

Figure 17 shows the testing process of the LLERR prototype. Plot (a) shows the volunteer wearing the LLERR lower limb exoskeleton with upright legs and holding double crutches as the initial state. Plot (b) shows the volunteer adjusting the position of the crutches, then the LLERR starting to plan the motion trajectory of the left-foot sole and backpropagating the target angle profile of the left (right) hip (knee) joint. Plot (c) shows the LLERR running the TD3 algorithm to control the left leg to complete the movement of the previous step, then planning the movement trajectory of the right-foot sole and backpropagating the target angle curve of the left (right) hip (knee) joint. Plot (d) shows the LLERR running the TD3 algorithm to control the right leg to complete the movement of the previous step. Plot (e) shows the crutches moving one step and next, the LLERR planning the trajectory of the left-foot sole and inversely extrapolating the target angle profile of the left (right) hip (knee) joint. Plot (f) shows the LLERR running the TD3 algorithm to control the left leg to complete the movement of the previous step. Plot (g) shows the crutch moving one step and the test completing.

The test results showed that the LLERR successfully completed the following tasks: planning the movement path of the sole, inferring the target angle curve of the hip (knee) joint, and controlling the hip (knee) joint motor to track the target angle curve. These operations enabled the LLERR to guide the volunteer through the process of walking up the stairs. Therefore, the LLERR can effectively assist patients with lower-limb motor rehabilitation in a staircase.

**Figure 18:**
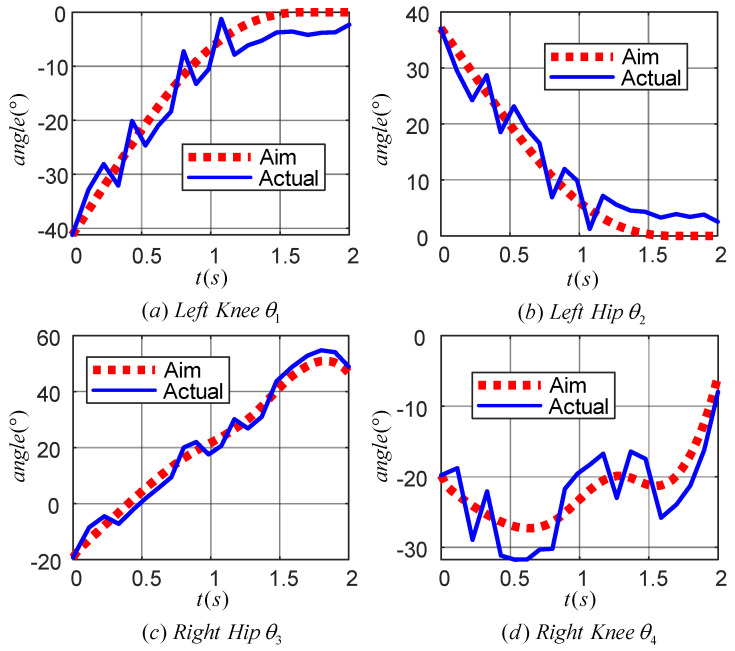
Joint angle tracking curve of the prototype lower-limb exoskeleton rehabilitation robot.

Figure 18 displays the motion curves of the hip (knee) joint angle of the LLERR prototype in the staircase environment running one cycle. The red dotted line represents the target joint angle curve, while the blue solid line represents the actual output curve of the LLERR joint angle. Overall, the trend of the LLERR joint angles was consistent with the target curve, indicating that the TD3 algorithm could control the joints of the LLERR to move according to the target trajectory. However, based on local observations, the actual motion trajectories of each joint of the LLERR deviated from the target motion trajectories and differed from the simulation effect graph shown in Figure 13. Possible causes of the issue included the following: during the modelling process, only the motion of the lower limbs in the sagittal plane was considered. However, the actual LLERR moved in three-dimensional space, which introduced some errors into the modelling. Additionally, the application of the TD3 algorithm was carried out based on the model established in the mathematical model section, and it is difficult to achieve accurate control when the actual model of the LLERR does not exactly match the training model, resulting in a certain gap between the actual motion trajectory and the expected trajectory. Despite these deviations, from the perspective of the prototype’s operation, the LLERR could effectively assist volunteers in climbing the stairs, indicating the effectiveness of the TD3 algorithm.

**Figure 19:**
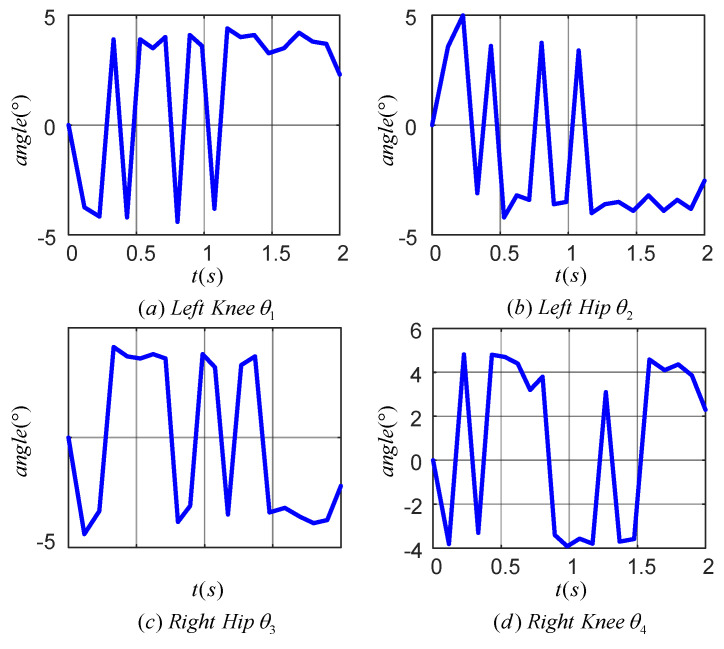
Joint tracking error of lower limb exoskeleton rehabilitation robot prototype.

Figure 19 displays the tracking error for each joint during one cycle of the LLERR. The angular tracking errors for the hip and knee joints were all within 5°, but there was an increase in error compared to the 0.6° error in Figure 14. Looking at the error curves for each joint, the errors were all above 2°. This could be attributed to the motor’s response delay and imprecise control of the current in the motor torque mode. However, overall, these tracking errors were not significant and did not hinder the LLERR’s success in climbing the stairs. This demonstrated the TD3 algorithm’s efficacy from an error analysis perspective.

## 5. Conclusions

The aim of this study was to investigate the control of lower-limb joint movement of an LLERR in a staircase setting. Initially, a mathematical model was developed. The planning of motion for the right-foot sole was executed using the TD3 algorithm. The target motion curve of the hip (knee) joint was derived through the kinematic law, leading to successful control of the joint motion of the LLERR. Theoretical simulation experiments and prototype experiments illustrated that the motion trajectory of the LLERR corresponded with the target trajectory. Despite some errors, they did not impede the LLERR’s objective of assisting the patient in climbing the staircase.

The results presented in this paper have significant theoretical and practical implications for the design and application of LLERRs, providing valuable insights and references for future research. However, there are some limitations in this study. For example, the error in the experiment still needs to be further reduced. The robot only moved in the sagittal plane. Moreover, the applicability of the algorithm in complex environments needs to be further verified. Future research can further improve the algorithm and construct more accurate 3D dynamic models to enhance its stability and accuracy in practical applications. In addition, the motion control strategy of the LLERR in unpredictable environments will be explored to adapt to a wider range of rehabilitation application scenarios.

## Data Availability

The data presented in this study are available on request from the corresponding author.
